# Molecular Signatures Correlated With Poor IVF Outcomes: Insights From the mRNA and lncRNA Expression of Endometriotic Granulosa Cells

**DOI:** 10.3389/fendo.2022.825934

**Published:** 2022-02-28

**Authors:** Libing Shi, Xianjiang Wei, Bingbing Wu, Chunhui Yuan, Chao Li, Yongdong Dai, Jianmin Chen, Feng Zhou, Xiang Lin, Songying Zhang

**Affiliations:** ^1^Assisted Reproduction Unit, Department of Obstetrics and Gynecology, Sir Run Run Shaw Hospital, Zhejiang University School of Medicine; Key Laboratory of Reproductive Dysfunction Management of Zhejiang Province, Hangzhou, China; ^2^International Institutes of Medicine, the Fourth Affiliated Hospital, Zhejiang University School of Medicine, Yiwu, China; ^3^Department of Clinical Medicine, Zhejiang University City College School of Medicine, Hangzhou, China

**Keywords:** RNA sequencing, mRNA, lncRNA, ovarian endometriosis, granulosa cells, retrieved oocyte numbers, female infertility

## Abstract

The outcomes of *in vitro* fertilization (IVF) for endometriotic women are significantly worse than for patients without ovarian endometriosis (OEM), as shown by fewer retrieved oocytes. However, the exact pathophysiological mechanism is still unknown. Thus, we conducted a prospective study that analyzed mRNA and lncRNA transcriptome between granulosa cells (GCs) from patients with fewer retrieved oocytes due to OEM and GCs from controls with male factor (MF) infertility using an RNA sequencing approach. We found a group of significantly differentially expressed genes (DEGs), including *NR5A2*, *MAP3K5*, *PGRMC2*, *PRKAR2A*, *DEPTOR*, *ITGAV*, *KPNB1*, *GPC6*, *EIF3A*, and *SMC5*, which were validated to be upregulated and negatively correlated with retrieved oocyte numbers in GCs of patients with OEM, while *DUSP1* demonstrated the opposite. The molecular functions of these DEGs were mainly enriched in pathways involving mitogen-activated protein kinase (MAPK) signaling, Wnt signaling, steroid hormone response, apoptosis, and cell junction. Furthermore, we performed lncRNA analysis and identified a group of differentially expressed known/novel lncRNAs that were co-expressed with the validated DEGs and correlated with retrieved oocyte numbers. Co-expression networks were constructed between the DEGs and known/novel lncRNAs. These distinctive molecular signatures uncovered in this study are involved in the pathological regulation of ovarian reserve dysfunction in OEM patients.

## Introduction

Endometriosis is defined as the presence of endometrial-like tissue outside of the uterine cavity, commonly proliferating onto the peritoneum and abdominal organs. This causes chronic inflammation with the formation of adhesions and is associated with pelvic pain and infertility (1, 2). Ovarian endometriosis (endometrioma, OEM), as one subtype of endometriosis, is formed by an intraovarian hematoma surrounded by ovarian cortex caused by recurrent ectopic endometrial hemorrhages and is present in up to 17%–44% of patients with endometriosis (3–5). Repeated and periodic bleeding of endometriotic lesions causes severe fibrosis and adhesions around the endometriotic cysts, leading to the destruction of the normal ovarian structure and reduction in the ovarian reserve (6).

*In vitro* fertilization (IVF) is recommended as an effective approach for infertility associated with endometriosis (7). However, the outcomes of IVF cycles for endometriotic women are significantly worse than for patients without this condition, as shown by the reduced numbers of oocytes retrieved (8–10) and attenuated oocyte quality (11, 12). Fewer mature follicles and retrieved oocytes reflect impaired folliculogenesis, which is the primary dilemma for women with OEM in attempting to achieve pregnancy. However, the exact pathophysiological mechanism of endometrioma related to lower numbers of retrieved oocytes is still unknown.

The GCs play an important role in regulating the development, maturation, and function of thecal cells and oocytes *via* signal transduction or direct cell-to-cell interactions, eventually affecting the whole process of follicle development, maturation, and atresia. Long noncoding RNAs (lncRNAs) are defined as transcripts that are longer than 200 nucleotides without significant protein coding potential (13, 14). The lncRNA and messenger RNAs (mRNAs) expression patterns were studied between eutopic and normal endometrium in the proliferative phase of OEM patients, which provided new resources for EM diagnosis and treatment (15–18). However, further analysis of mRNA and lncRNA profile and regulatory mechanism at GCs level has not been reported, nor has it been associated with infertility and IVF outcomes.

RNA sequencing (RNA-seq) allows the investigation of transcriptomes, including mRNA and lncRNA, at unsurpassed resolution of biological processes. RNA-seq analysis from oocytes and GCs during the stepwise follicular development reveals the transcriptomic landscape of folliculogenesis (19). To characterize the cellular heterogeneity of GCs and provide insights into the complex transcriptomes of GCs from patients with OEM, here, we aimed to identify the mRNA and lncRNA transcriptional regulation landscape of GCs from patients with OEM using an RNA-seq approach.

## Results

### Differential mRNA Transcriptional Signatures of GCs Associated With OEM

GCs collected from infertile women due to OEM were put in the OEM group, while GCs from women in couples with male factor (MF) infertility were regarded as control. The clinical characteristics of GC samples for RNA-seq, detailed in **Table 1**, showed that the only significantly different parameter between the two groups was the number of retrieved oocytes (*p* < 0.05).

**Table 1:** Clinical characteristics of infertile patients for GC RNA-seq.

Parameters	MF group (*N* = 6)	OEM group (*N* = 5)	*p*-value
Age (year)	28.83 ± 4.36	29.0 ± 2.45	0.94
BMI (kg/m^2^)	20.26 ± 2.48	19.63 ± 2.11	0.67
AMH (ng/ml)	4.5 ± 1.64	4 ± 1.41	0.61
Infertility duration (year)	3.0 ± 2.53	1.8 ± 0.84	0.32
Basal E2 level (ng/L)	48.0 ± 20.59	46.20 ± 25.40	0.90
Basal FSH level (IU/L)	8.33 ± 2.07	8.40 ± 2.70	0.96
Basal antral follicle count	9.83 ± 2.93	8.40 ± 4.39	0.53
The starting dosage of Gn (IU)	193.67 ± 41.84	227.6 ± 43.55	0.22
The total dosage of Gn (IU)	2322.83 ± 616.29	2535.0 ± 906.85	0.66
Days of Gn stimulation (day)	10.50 ± 2.26	10.60 ± 1.67	0.94
The dosage Gn per day (IU)	220.50 ± 27.52	233.4 ± 46.95	0.58
Retrieved Oocytes NO.	15.17 ± 4.71	9.0 ± 3.24	0.04*
Growing follicle NO.	10.33 ± 4.63	9.2 ± 3.35	0.66
2PN zygote NO.	10.17 ± 3.76	3.65 ± 3.78	0.18
Cleavage stage embryo NO.	9.83 ± 3.31	7.20 ± 2.68	0.19
High qualified embryo NO.	3.67 ± 2.16	3.60 ± 1.67	0.96
High qualified embryo rate	37.29% (22/59)	50.0% (18/36)	0.22
2PN fertilization rates	73.49% (61/83)	80% (36/45)	0.41

Mean ± SD for continuous variables was given. Continuous variables were compared using the Student’s t-test. The rates for comparison were analyzed using chi-square test.

N, sample size; GCs, granulosa cells; RNA-seq, RNA sequencing; MF, male factor; OEM, ovarian endometriosis; BMI, body mass index; AMH, anti-Müllerian hormone; E2, estradiol; FSH, follicle-stimulating hormone; Gn, gonadotropins; NO., number; 2PN, two-pronuclear. (*p < 0.05).

**Figure 1:**
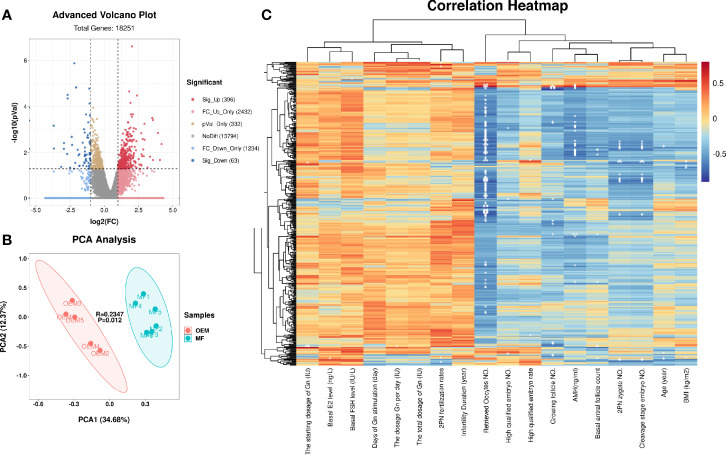
mRNA profiling of granulosa cells (GCs) from patients with infertility associated with ovarian endometriosis (OEM). **(A)** The x-axis of the volcano map represents log_2_(fold change (FC)) value of total detected genes, while the y-axis represents their −log_10_(*p* value) value. The significant FC_up differentially expressed genes (DEGs) are presented in the upper right quadrant (red), and the significant FC_down DEGs are shown in upper left quadrant (blue). **(B)** Two-dimensional principal component analysis (PCA) of DEGs from the transcriptomes of RNA-seq data. The PC1 separates the two groups (MF versus OEM). The PC2 separates the samples according to the gene expression pattern. Green color indicates samples from the male factor (MF) group, and red color indicates samples from the OEM group. **(C)** Correlation heatmap between DEGs and the clinical characteristics of RNA-seq GC samples. The ordinate represents DEGs, and the abscissa is clinical characteristics (*p < 0.05, **p < 0.01, ***p < 0.001) (*N*_MF_ = 6, *N*_OEM_ = 5).

The quality control statistics for the RNA-sequencing is presented in **Supplementary Table S1**. The RNA quality detection data of GCs for RNA-seq samples are presented in **Supplementary Table S2** and **Supplementary Figures S1A–C**. The expression of cell-type-specific markers for GCs is shown in **Supplementary Figures S1D–O**. There were 15,890 mRNAs detected in total, of which 459 significantly DEGs (396 upregulated and 63 downregulated; **Figure 1A** and **Supplementary Table S3**) were found in the GCs by comparing the two groups. Z-score normalized fragments per kilobase of transcript per million mapped reads (FPKM) of differentially expressed genes were used as inputs for two-dimensional principal component analysis (PCA) by using R package Stats (**Figure 1B**). There was a clear separation between the distribution of the GC mRNA datasets from the OEM and MF group (*p* < 0.05).

To clarify the correlation between the expression of DEGs and the clinical characteristics of samples, we constructed their correlation heatmap (**Figure 1C**). The results showed that most of the DEGs were significantly correlated with the number of retrieved oocytes, followed by 2PN zygote numbers, cleavage stage embryo numbers, and AMH level.

### Functional Enrichment Analysis of GCs From the OEM Group Compared With the MF Group

**Figure 2:**
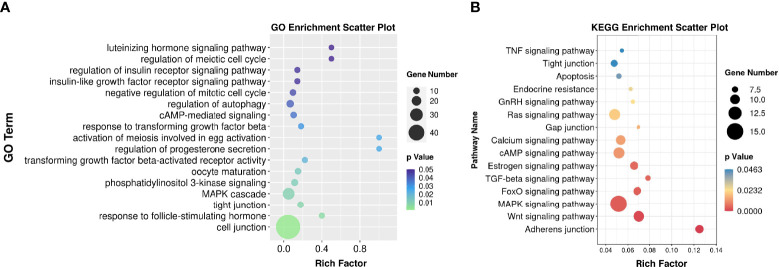
Functional enrichment analysis. **(A)** Gene Ontology (GO) bubble chart for DEG enrichment. **(B)** Kyoto Encyclopedia of Genes and Genomes (KEGG) bubble chart indicating the enrichment of DEGs. Rich factor is defined as S gene number divided by B gene number. “S” was annotated as the number of genes with significant difference in specific GO/KEGG categories; “B” was the number of genes for a specific GO/KEGG (*N*_MF_ = 6, *N*_OEM_ = 5).

Functional enrichment analysis of these DEGs helped to identify the pathways that affected the function of GCs from patients with OEM. GO analysis demonstrated that these DEGs were mainly involved in the following terms: cell junction, MAPK cascade, oocyte maturation, responses to FSH and LH, and cell cycle (**Figure 2A**). By using KEGG analysis, we found that these DEGs were involved in several pathways, including adhesion junction, Wnt signaling, MAPK signaling, FoxO signaling, cAMP signaling, and estrogen signaling pathway (**Figure 2B**).

### OEM-Associated DEGs Screen for Validation

**Figure 3:**
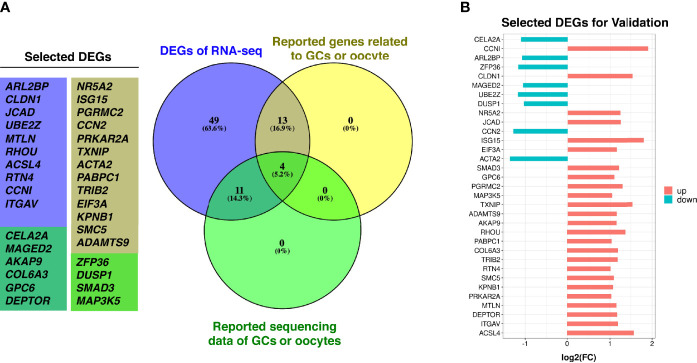
DEGs selection for validation. **(A)** Purple circle represents DEGs with |log_2_(FC)| ≥ 1, *p* < 0.08 and FPKM > 0.8 in all samples from this study, yellow circle represents reported genes from the DEGs that are related to GCs/oocyte/follicular development, green circle represents genes from the DEGs are intersected with reported original transcriptomic sequencing data of GCs and OEM-affected oocytes. **(B)** The log_2_(FC) value of selected DEGs for validation (*N*_MF_ = 6, *N*_OEM_ = 5).

In order to screen more potential DEGs for validation, we included the mRNA with threshold of |log_2_(fold change (FC))| ≥ 1, *p* < 0.08, and FPKM >0.8 in all samples; then, 77 mRNA were selected. Among them, 60 genes were upregulated, and 17 genes were downregulated simultaneously. To validate the transcriptomic sequencing results, genes that were previously reported related to GCs/oocyte/follicular development, genes that intersected with reported original transcriptomic sequencing data for GCs (19) and OEM-affected oocytes (20), and genes that were involved in significantly enriched GO functions of this study were selected from the significant 77 DEGs and tested on more clinical GC samples (**Figure 3A** and **Supplementary Table S4**). These included up- and downregulated genes demonstrated in **Figure 3B**.

### Validation of DEGs and Correlation With IVF Clinical Outcomes

The clinical characteristics of patients’ cohort for validation are listed in **Table 2**. The average AMH, basal antral follicle count, retrieved oocytes number, growing follicle number, 2PN zygote number, cleavage stage embryo number, high qualified embryo number, and 2PN fertilization rates of OEM group were also significantly lower than those in MF group, while basal FSH level was also higher. These clinical manifestations are consistent with the phenomenon that OEM leads to the ovarian reserve dysfunction and compromised oocyte quality.

**Table 2:** Clinical characteristics of infertile patients for validation.

Parameters	MF group (*N* = 46)	OEM group (*N* = 42)	*p*-value
Age (year)	29.78 ± 4.17	32.64 ± 4.32	0.02*
BMI (kg/m^2^)	21.43 ± 2.19	21.01 ± 2.17	0.37
AMH (ng/ml)	4.53 ± 2.81	2.23 ± 2.01	<0.001***
Infertility duration (year)	3.09 ± 2.40	3.55 ± 3.63	0.57
Basal E2 level (ng/L)	37.53 ± 16.11	37.66 ± 17.79	0.97
Basal FSH level (IU/L)	6.23 ± 2.54	7.71 ± 3.40	0.03*
Basal antral follicle count	10.04 ± 4.58	6.24 ± 3.98	<0.001***
The total dosage of Gn (IU)	2050.0 ± 573.67	2446.73 ± 1061.73	0.14
Days of Gn stimulation (day)	9.67 ± 1.80	9.59 ± 2.46	0.85
Retrieved oocytes NO.	11.09 ± 6.14	5.71 ± 4.19	<0.001***
Growing follicle NO.	10.15 ± 4.07	6.38 ± 3.41	<0.001***
2PN zygote NO.	6.87 ± 4.72	3.38 ± 2.73	<0.001***
Cleavage stage embryo NO.	6.54 ± 4.64	3.24 ± 2.68	<0.001***
High qualified embryo NO.	2.39 ± 2.15	1.19 ± 1.71	0.004**
High qualified embryo rate	36.54% (110/301)	36.76% (50/136)	0.96
2PN fertilization rates	68.70% (316/460)	61.21% (142/232)	0.049*

Mean ± SD for continuous variables was given. Continuous data with a normal distribution, parameters were compared using Student’s t-test, while non-normally distributed parameters were compared using the Mann–Whitney nonparametric U-test. The rates for comparison were analyzed using chi-square test.

N, sample size; GCs, granulosa cells; MF, male factor; OEM, ovarian endometriosis; BMI, body mass index; AMH, anti-Müllerian hormone; E2, estradiol; FSH, follicle-stimulating hormone; Gn, gonadotropins; NO., number; 2PN, two-pronuclear. (*p < 0.05, **p < 0.01, ***p < 0.001).

**Figure 4:**
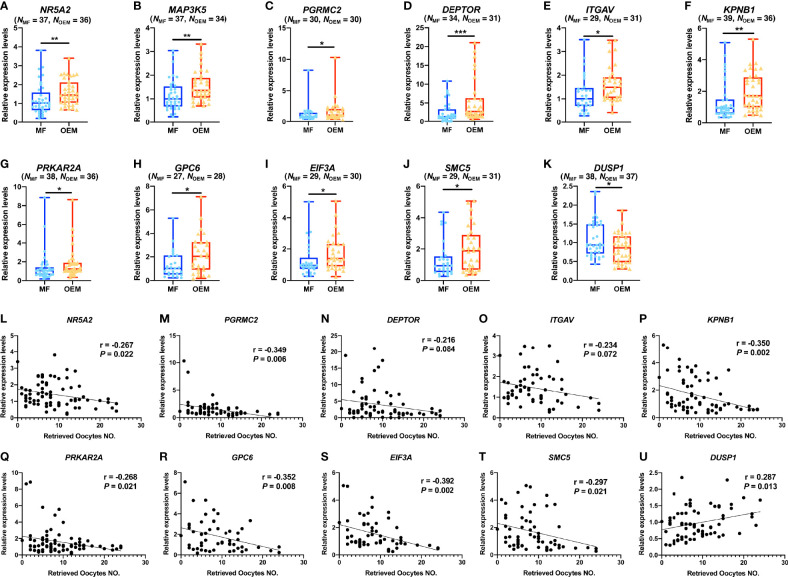
OEM-associated DEGs validation and correlation analysis with retrieved oocyte numbers. **(A–K)** Validation of selected DEGs by expanding clinical samples through real-time quantitative polymerase chain reaction (qPCR) amplification demonstrate significant differences between the OEM and MF groups. (N, sample size). **(L–U)** Pearson correlation analyses between relative expression levels of the validated DEGs and the retrieved oocyte numbers (r, Pearson correlation coefficient). (*p < 0.05, **p < 0.01, ***p < 0.001).

The quantitative PCR (qPCR) results of GCs for validation showed that the relative expressions of *NR5A2* (**Figure 4A**), *MAP3K5* (**Figure 4B**), *PGRMC2* (**Figure 4C**), *DEPTOR* (**Figure 4D**), *ITGAV* (**Figure 4E**), *KPNB1* (**Figure 4F**), *PRKAR2A* (**Figure 4G**), *GPC6* (**Figure 4H**), *EIF3A* (**Figure 4I**), and *SMC5* (**Figure 4J**) were indeed significantly higher in OEM group when compared with the MF group, while *DUSP1* (**Figure 4K**) was lower expressed in OEM group.

We analyzed the correlations of the qPCR value with the IVF clinical outcomes, including the numbers of retrieved oocytes, 2PN zygote, cleavage stage embryo on day 3 of culture (developed from 2PN zygotes), and growing follicles (diameter ≥ 14 mm on the day of hCG triggering), in validation cohort. Our results demonstrated that, among the validated DEGs, the number of retrieved oocytes was negatively correlated with the relative gene expression levels of *NR5A2* (**Figure 4L**), *PGRMC2* (**Figure 4M**), *DEPTOR* (**Figure 4N**), *ITGAV* (**Figure 4O**), *KPNB1* (**Figure 4P**), *PRKAR2A* (**Figure 4Q**), *GPC6* (**Figure 4R**), *EIF3A* (**Figure 4S**), and *SMC5* (**Figure 4T**) and positively correlated with the relative expression of *DUSP1* (**Figure 4U**). In addition, the number of 2PN zygote (**Supplementary Figures S2A–J**), cleavage stage embryo (**Supplementary Figures S3A–J**), and growing follicles (**Supplementary Figures S4A–G**) were correlated with the relative gene expression levels of the validated DEGs in this study.

### lncRNA Profiling of GCs From the OEM or MF Patient Groups

**Figure 5:**
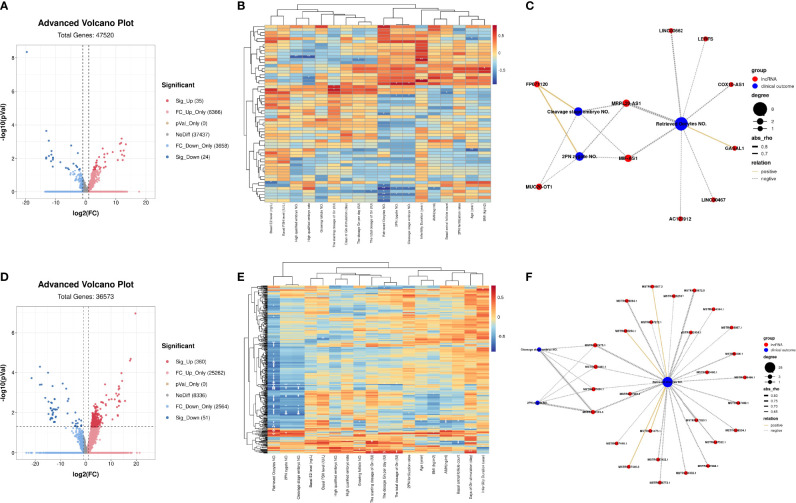
LncRNA profiling of GCs from patients with infertility associated with OEM. **(A)** Volcano map analysis of differentially expressed known lncRNA indicates significantly upregulated lncRNAs (red), downregulated significantly lncRNAs (blue), and non-significant lncRNAs (gray). **(B)** Correlation heatmap between differentially expressed known lncRNA and the clinical characteristics of RNA-seq samples. The ordinate represents known lncRNA, and the abscissa is clinical characteristics. **(C)** Co-expression network of clinical characteristics and known lncRNAs through Weighted Gene Co-expression Network Analysis (WGCNA); Cytoscape was used to visualize the network diagram (|correlation coefficient| > 0.5, *p* < 0.05). **(D)** Volcano map analysis of differentially expressed novel lncRNA. **(E)** Correlation heatmap between differentially expressed novel lncRNA and the clinical characteristics of RNA-seq samples. **(F)** Co-expression network of clinical characteristics and novel lncRNAs through WGCNA; Cytoscape was used to visualize the network diagram (|correlation coefficient| > 0.5, *p* < 0.05) (*p < 0.05, **p < 0.01, ***p < 0.001) (*N*_MF_ = 6, *N*_OEM_ = 5).

Through RNA-seq, we detected 47,414 non-protein coding transcripts from these GC samples. Among these transcripts, there were 14,379 known lncRNA annotated in the genome, and 33,035 novel lncRNAs were predicted. Differential expression analysis demonstrated that 35 known lncRNAs were significantly upregulated including PTPRG-AS1, LINC01278, MEG8, LERFS, PRKCQ-AS1, and SNHG17, and 24 known lncRNAs were significantly downregulated including MALAT1, FTX, BDNF-AS, DANCR, and GASAL1 (**Figure 5A** and **Supplementary Table S3**). The correlation heatmap showed that most of the differentially expressed known lncRNA were also related to the number of retrieved oocytes, followed by 2PN zygote numbers and cleavage stage embryo numbers (**Figure 5B**). Interestingly, the correlation network diagram showed that most of significantly differentially expressed known lncRNAs were negatively correlated with these clinical indicators. For example, MRPL20-AS1 and MIF-AS1 were all negatively correlated with the number of retrieved oocytes, 2PN zygote numbers, and cleavage stage embryo numbers (**Figure 5C**).

For the predicted novel lncRNA, 360 of them were significantly upregulated, and 51 were significantly downregulated (**Figure 5D** and **Supplementary Table S3**). The correlation heatmap also showed that most of the differentially expressed novel lncRNAs were also related to the number of retrieved oocytes, followed by 2PN zygote numbers and cleavage stage embryo numbers (**Figure 5E**). Meanwhile, through screening, in the novel lncRNA whose average FPKM of all samples was ≥1, we found that most of these lncRNA were negatively correlated with these clinical characteristics. Novel lncRNA, such as MSTRG.47475.1, MSTRG.51840.1, MSTRG.47454.1, and MSTRG.14101.4 were all negatively expressed with the number of retrieved oocytes, 2PN zygote numbers, and cleavage stage embryo numbers (**Figure 5F**).

### mRNA–lncRNA Co-expression Networks Establishment

**Figure 6:**
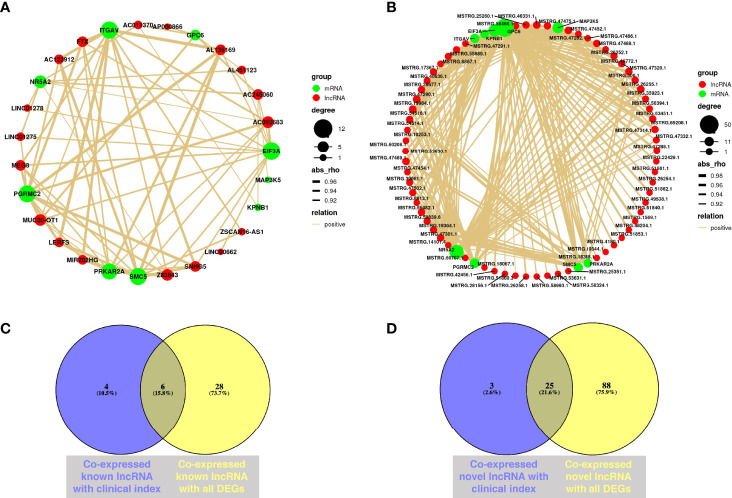
Co-expression analysis of mRNA and lncRNA. **(A)** Co-expression network from validated DEGs and known lncRNAs through WGCNA; Cytoscape was used to visualize the network diagram (|correlation coefficient| > 0.9, *p* < 0.05). **(B)** Co-expression network from validated DEGs and novel lncRNAs through WGCNA; Cytoscape was used to visualize the network diagram (|correlation coefficient| > 0.9, *p* < 0.05). **(C)** Venn diagram of co-expressed known lncRNAs with clinical characteristics and validated DEGs. Purple circle represents co-expressed known lncRNA with clinical characteristics, while yellow circle means co-expressed known lncRNA with all validated DEGs (|correlation coefficient| > 0.5, *p* < 0.05). **(D)** Venn diagram of co-expressed novel lncRNAs with clinical characteristics and validated DEGs. Purple circle represents co-expressed novel lncRNA with clinical characteristics, while yellow circle means co-expressed novel lncRNA with all validated DEGs (|correlation coefficient| > 0.5, *p* < 0.05) (*N*_MF_ = 6, *N*_OEM_ = 5).

Establishing mRNA–lncRNA regulatory networks is useful for revealing interactions between mRNAs and co-expressed lncRNAs. We identified 210 significant mRNA–lncRNA(known) groups with correlation coefficient threshold |abs_rho| > 0.5 and *p* < 0.05. Most of them were (208/210) positively associated, and only two of the groups were negatively associated. The validated DEGs, including *NR5A2*, *MAP3K5*, *PGRMC2*, *ITGAV*, *KPNB1*, *PRKAR2A*, *GPC6*, *EIF3A*, and *SMC5* co-expressed with known lncRNAs under the correlation coefficient threshold |abs_rho| > 0.9 and *p* < 0.05 (**Figure 6A**). Among them, *EIF3A*, *PGRMC2*, *ITGAV*, *SMC5*, and *PRKAR2A* had over 10 paired co-expressed known lncRNA (**Supplementary Table S5**).

Simultaneously, we identified 750 mRNA–lncRNA(novel) groups with correlation coefficient threshold |abs_rho| > 0.5 and *p* < 0.05 from the novel lncRNA with average FPKM value of all samples ≥ 1. Most of them were (744/750) positively associated, while only six of the groups were negatively associated. Identically, under the correlation coefficient threshold |abs_rho| > 0.9 and *p* < 0.05, validated DEGs including *NR5A2*, *MAP3K5*, *PGRMC2*, *ITGAV*, *KPNB1*, *PRKAR2A*, *GPC6*, *EIF3A*, and *SMC5* had co-expressed novel lncRNAs, while *DEPTOR* and *DUSP1* had not (**Figure 6B**). Among them, *GPC6*, *NR5A2*, *EIF3A*, and *MAP3K5* had over 20 pairs of co-expressed novel lncRNA (**Supplementary Table S5**).

The Venn diagrams demonstrated that MIF-AS1, LERFS, AC123912, LINC00662, MUC20-OT1, and FP671120 were co-expressed known lncRNAs, which both correlated with clinical characteristics and validated DEGs (**Figure 6C**). Novel lncRNAs, such as MSTRG.15872.5, MSTRG.47475.1, MSTRG.19364.1, MSTRG.14101.4, and MSTRG.42454.1, were all associated with clinical characteristics and validated DEGs (**Figure 6D**).

## Discussion

Oocytes, granulosa cells (GC), and follicular fluid are the main components of follicles. GCs grow closely around the oocyte, constitute the microenvironment for oocyte survival, and play an important role in follicle and oocyte health (21). The direct exchanging of small molecules *via* gap junction channels and bidirectional paracrine signaling between oocytes and GCs are critical to synchronize follicle development and oocyte maturation, ovulation, and atresia (22). On the other hand, redundant GCs would be discarded in the process of oocyte retrieval; their acquisition is non-invasive to human body nor to oocytes. Furthermore, many studies have correlated GCs gene expression with different outcome measures such as oocyte maturity, fertilization, embryo development, implantation, and pregnancy (23–25). GCs transcriptome has also been studied to identify gamete quality (26) and explore the potential of clinical implications for the treatment of infertility (27). Therefore, we choose GCs as a noninvasive indicator of oocytes competence and ovarian function.

In the clinical characteristics of the validation cohort in **Table 2**, the mean age of patients in OEM group was 3 years older than that in the MF group, although the average age of both groups was below 35 years old. On the one hand, for these patients enrolled in the MF group, the female partners are generally healthy, they do not need extra surgical treatment before getting pregnant. However, for these patients with OEM, all of them underwent previous laparoscopic surgery. Thus, the time of IVF treatment is delayed. Due to the poor ovarian reserve, the number of IVF treatment cycles is much more than that of the MF group. On the other hand, the clinical indexes about ovarian reserve, such as AMH level, were dramatically decreased in OEM group, which was far more than the fecundity drops caused by advancing age from 30 to 33 years old. There are two main possible reasons for the significant decline of ovarian reserve in OEM patients, rather than age in our cohort. One is the space occupation of OEM cyst, and subsequent ovarian tissue fibrosis reduces the functional ovarian tissue (6, 28). The other is that inevitable removal of unaffected ovarian tissue during the surgical excision of endometrioma cyst wall aggravates the reduction in ovarian reserve function (3, 29). Therefore, it is the dual factors of OEM itself and laparoscopic surgery that lead to the significant decrease in ovarian reserve.

GCs act under steroid hormonal control and play key roles in folliculogenesis and corpus luteum formation after ovulation. The DEGs we identified in this study, including *NR5A2* and *PGRMC2*, are involved in steroid hormone receptor activity and steroid hormone-mediated signaling pathways. *NR5A2* plays pivotal roles in the regulation of steroidogenic enzymes (30) and is essential for progesterone synthesis (31) and luteinization (32). The high expression of *NR5A2* in our study indicates the increased ability of GCs from patients with OEM to synthesize and secrete progesterone, with possible premature luteinization of follicles and follicle atresia (33) and reflecting reduced ovarian reserve (34, 35). Moreover, *PGRMC2*—coding for progesterone receptor membrane component 2—was reported to be involved in ovarian folliculogenesis (36–38). An increased expression of *PGRMC2* in patients was reported to be associated with diminished ovarian reserve (39).

Follicular atresia is mostly indicated by GC apoptosis (33). *MAP3K5*, a significant DEG validated in this study, is a mediator of the MAPK pathway, also known as apoptosis signal regulating kinase 1 (ASK1) (40). *MAP3K5* was reported to suppress healthy rabbit GC functions, including promoting apoptosis, inhibiting proliferation, and altering progesterone release (41). Moreover, *MAP3K5* is involved in intrinsic apoptotic signaling pathway in response to oxidative stress through GO evaluation. Excessive oxidative stress is reported to occur in GCs (12, 42), and in follicular fluid (43), from patients with OEM and is associated with infertility. In addition, another validated *DEG*, *KPNB1*, interacts with *MAP3K5* in response to stress (44) and plays a role in mouse oogenesis and folliculogenesis (45). The upregulated expression of *MAP3K5* and *KPNB1* suggests that they may be involved in the apoptosis of GCs, which is consistent with the study that GCs from patients with endometriosis show increased levels of apoptosis (46).

The delicate interactions between GCs and oocytes through cell junctions are essential for follicular development and in ensuring oocyte competence. Abnormal GC function can reflect incompetent oocytes. *PRKAR2A* plays an important role in the maturation of GCs (47) and also has critical functions in meiotic arrest and meiotic maturation in mammalian oocytes (48, 49). *SMC5*, together with *SMC6*, functions in DNA repair (50) and in successful meiotic divisions (51, 52). Thus, the upregulated expression levels of *PRKAR2A* and *SMC5* in GCs from patients with OEM in this study are likely to reflect failure of meiotic resumption.

Inhibition of *DUSP1* in cumulus cells caused abnormal cell cycle progression (53). RNA-seq of oocytes from OEM patients revealed that *DUSP1* might be a potential biomarker to diagnose oocyte quality due to their association with decreased oocyte competence (20). In our study, *DUSP1* was downregulated in OEM group, and its relative expression levels were positively correlated with retrieved oocyte number. This suggests that *DUSP1* may play a role in the process of OEM affecting follicular development.

LncRNAs in GCs are not only largely involved in normal folliculogenesis, but their function and expression will change accordingly under pathological conditions (54). LncRNA MALAT1 is one of the studied lncRNAs in endometriosis. It mediates hypoxia-induced pro-survival autophagy of endometrial stromal cells in endometriosis (55), inhibits apoptosis of endometrial stromal cells by activating PI3K-AKT pathway (56), and involves in the E2-mediated epithelial–mesenchymal transition process in endometriosis (57). Our results are consistent with the reported study, which demonstrated that lncRNA MALAT1 was significantly downregulated in GCs from OEM patients (58). The downregulation of lncRNA MALAT1 in GCs may have an adverse effect on the growth and development of oocytes by inhibiting GC proliferation through MAPK pathway. Interestingly, two of these predicted upregulated novel lncRNAs, namely,

MSTRG.13411.12 and MSTRG.13411.14 are near MALAT1 locus, suggesting that MALAT1 is involved in the pathological regulation of GCs from OEM-related infertility patients.

The known lncRNA, Z83843, an autophagy-related lncRNA (59), co-expresses with *NR5A2* and *PGRMC2*. *PRKAR2A*, *SMC5*, *ITGAV*, *PGRMC2*, and *EIF3A* have more than eight overlapped co-expressed known lncRNA, including MEG8, Z83843, AC092683, MUC20-OT1, FTX, SNHG5, AL136169, and LINC01275. In our study, MEG8 is one of the most significantly upregulated lncRNAs, while FTX is significantly downregulated in GCs from OEM patients. *EIF3A* also correlated with LINC00662 and LINC01278, which are reported to be involved in many biology functions, such as cell proliferation (60), carcinoma metastasis (61), and M2 macrophage polarization *via* activating Wnt/β-catenin signaling (62).

Furthermore, LINC00662 is correlated with oocyte-retrieved number and the expression of *EIF3A*. LINC00662 is reported to be involved in regulating oxidative stress (63); also, it plays a role in spermatogenesis and male infertility (64). MUC20-OT1 is highly correlated with 2PN zygote number and the expression of *PRKAR2A*, *GPC6*, *SMC5*, *EIF3A*, *ITGAV*, and *PGRMC2*. AC123912 is highly correlated with oocyte-retrieved number and the expression of *SMC5*, *EIF3A*, *PRKAR2A*, *ITGAV*, and *PGRMC2*. LERFS is a known lncRNA that is highly correlated with oocyte-retrieved number and the expression of *SMC5*, *PRKAR2A*, *ITGAV*, and *PGRMC2*. The function of these known lncRNAs were mainly studied in cancer research (62, 65). On the other hand, many of the novel lncRNAs that are associated with clinical characteristics and validated DEGs, such as MSTRG.47489.1, MSTRG.31476.1, and MSTRG.58466.1, are located near LSAMP locus, which indicates that these novel lncRNAs may play a role in transcriptional regulation by targeting at LSAMP.

This study creatively demonstrated the transcriptional regulation profile of GCs related to OEM, screened out the mRNAs and lncRNAs that correlated with the low retrieved oocyte number due to OEM, and mapped their co-expression network. It provides predictive value for oocyte competence through GC gene diagnosis. However, the limitation of this study is lacking of in-depth mechanism research of those mRNAs and lncRNAs, which deserve to be further studied on their specific biological functions in our future work. In addition, the sample size of each group for RNA-seq is <10 cases, which might inevitably lead to incompleteness of gene screening and bias of data analysis.

In conclusion, our results have given a better understanding for OEM in the transcriptomic level. The major findings are as follows (1): we show a differential mRNA and lncRNA expression profiling of GCs from OEM patients and report significant differentially expressed mRNAs, known and novel lncRNAs; (2) we have identified a group of mRNAs and lncRNAs that likely to be involved in regulating ovarian reserve and are closely correlated to IVF clinical outcomes; and (3) we have constructed mRNA–lncRNA co-expression networks that display abnormal and complex regulatory mechanisms of GCs from infertility patients due to OEM at transcriptional level. These findings demonstrate that the distinctive molecular signatures are involved in the pathological process of ovarian reserve dysfunction in OEM patients, and the potential regulatory relationship between mRNA and lncRNA in GCs from OEM patients make some novel lncRNAs as diagnostic or therapeutic targets.

## Materials and Methods

### Ethical Approval

The use of human GC samples for this study was approved by the Institutional Review Board of Sir Run Run Shaw Hospital, Zhejiang University School of Medicine (ethical approval no. 20200423-44), and signed informed consent was obtained from all participants before inclusion.

### Patient Selection

GCs were collected from infertile women who underwent IVF due to unilateral or bilateral OEM or from women in couples with MF infertility (henceforth “MF group”) from May 2018 to April 2019. The inclusion criteria for both groups of women included an age of 20–42 years, regular menstrual cycles of 25–35 days, body mass index (BMI) of 18–25 kg/m^2^, and normal hormone profile in the early proliferative phase (days 2–5 of the menstrual cycle). Furthermore, patients from the MF group did not show signs of endometriosis or any other female infertility problems. For these patients, the main reason of infertility was diagnosed low sperm quantity and quality, abnormal chromosome, or sexual dysfunction of their husband. Simultaneously, patients from the OEM group underwent previous laparoscopic surgery for unilateral or bilateral ovarian endometriomas, with clear pathological diagnosis, and were classified as stage II–IV according to the World Endometriosis Society consensus on the classification of endometriosis (2). The detailed information about the distribution (bilaterally or unilaterally), location, and size of lesions of all OEM patients included in this study is listed in **Supplementary Table S6**. In addition, patients with chromosomal abnormalities, acute inflammation, malignant tumors of the reproductive system, previous endometrial neoplasias, or other anatomical endometrial pathologies were excluded.

### Ovarian Stimulation and GC Collection

All patients included in this study underwent controlled ovarian stimulation with gonadotropins. Oocyte retrieval was scheduled 36 h later after human chorionic gonadotropin (hCG, Livzon, Guangdong, China) was administered. When preovulatory follicles were aspirated, the oocyte–cumulus complexes were pulled from the follicle wall with the fluid. Under a microscope, all the oocyte–cumulus complexes from one single patient were identified and picked out and then washed in Gemmate medium (Cook Medical, Indianapolis, IN, USA). Before oocytes were removed for further culture prior to IVF, oocytes and cumulus cells were separated from the oocyte–cumulus complexes with syringe needles. The rest of the cumulus cells, as a subtype of GCs, were pooled and collected for RNA extraction.

### RNA-seq Library Construction

Total RNA was extracted using Trizol reagent (Invitrogen, Carlsbad, CA, USA) following the manufacturer’s procedure. The total RNA quantity and purity were analyzed using an Agilent Bioanalyzer 2100 and RNA 1000 Nano LabChip kits (Agilent Technologies, Santa Clara, CA, USA) with an RNA integrity number >7.0. An RNA-seq library was constructed following the manufacturer’s instructions using SMARTer^®^ Stranded Total RNA-Seq kits v. 2 (Takara Bio USA, Inc., Mountain View, CA, USA). The first-strand cDNA was synthesized from 10 ng of diluted RNA. Then, Illumina adapters and barcodes were added to the cDNA fragments. After purification of the RNA-Seq library with AMPure beads and depletion of ribosomal cDNA with ZapR v. 2 and R-Probes v. 2, the final RNA-Seq library was amplificated and enriched. The mean insert size for the paired-end libraries was 300 bp (± 50 bp).

### Sequencing and Primary Analysis

Paired-end sequencing was performed on an Illumina X10 sequencing system with a constructed human GC RNA-seq library at LC-Bio Technology Co. Ltd. (Hangzhou, China). The average sequencing depth is 10.98 G. After quality control for reads, 5.81 G was left.

### Expression Analysis

We aligned reads to the UCSC *Homo sapiens* reference genome GRCh38 (http://genome.ucsc.edu/) using the HISAT package (http://daehwankimlab.github.io/hisat2/). HISAT allows multiple alignments per read (up to 20 by default) and a maximum of two mismatches when mapping the reads to the reference genome.

The mapped reads of each sample were assembled using StringTie (https://ccb.jhu.edu/software/stringtie/). Then, all transcriptomes from the samples were merged to reconstruct a comprehensive transcriptome using gffcompare (github.com/gpertea/gffcompare/). StringTie (66) was used to calculate the expression levels for mRNAs and lncRNAs by calculating FPKM. The differentially expressed mRNAs and lncRNAs were selected with |log_2_(FC)| ≥ 1 and with statistical significance (*p* < 0.05) using R package edgeR (67).

### LncRNA identification

To identify novel lncRNA, transcripts that overlapped with known mRNAs and transcripts shorter than 200 bp were discarded. Then, we utilized CPC0.9-r2 (68) (cpc2.cbi.pku.edu.cn/) with default parameters (cpc2 -i novel.fa -o cpc2.out) and CNCI2.0 (69) (www.bioinfo.org/software/cnci) with default parameters (CNCI.py -f novel.fa -o CNCI.result -p 1 -m ve -g novel.gtf -d genome.fa) to predict the coding potential for transcripts. All transcripts with CPC score <−1 and CNCI score <0 were removed, and the remained transcripts were considered as novel lncRNAs.

### Functional Enrichment Analysis

All the significantly DEGs, including 396 upregulated and 63 downregulated genes, were subjected to Kyoto Encyclopedia of Genes and Genomes (KEGG) enrichment and Gene Ontology (GO) evaluation. “S” was the number of genes with significant difference in specific GO/KEGG categories, “TS” represented the number of genes with significant difference, “B” was the number of genes for a specific GO/KEGG, and “TB” was the total number of genes. KEGG pathways enriched and presented downregulated and upregulated genes with similar biological function. The enrichment of down- and upregulated genes for the same GO function was also demonstrated. The higher rich factor (S gene number divided by B gene number) represents the higher enrichment degree. The calculation formula of *p*-values of enrichment analysis was: $P=1-\sum_{i=0}^{S-1}\frac{{(}_{i}^{B}){(}_{TS-i}^{TB-B})}{{(}_{TS}^{TB})}$ . The R package Ggplot2 (70) was used to visualize the data of analysis.

### Validation

Selected DEGs were corroborated through real-time quantitative polymerase chain reaction (qPCR) as described previously (6). Primers are shown in **Supplementary Table S7**. The validation of primers for qPCR was estimated through the melting curve of qPCR procedure. Glyceraldehyde 3-phosphate dehydrogenase (GAPDH) was used as the endogenous control. For each sample, the average cycle threshold (Ct) was calculated from triplicate wells that varied by <0.5 Ct. All Ct values were normalized against the average Ct values of the GC samples from the MF patient group.

### Co-expression Network Analyses

The Weighted Gene Co-expression Network Analysis (WGCNA) (71) was used to obtain the network relationship. According to the relationship, Cytoscape was used to draw the network diagram. Correlation network was performed using the OmicStudio tools at https://www.omicstudio.cn/tool. A Pearson’s correlation analysis was subsequently performed to cluster samples and detect outliers. The threshold of co-expression network between known/novel lncRNAs and clinical characteristics was with |correlation coefficient| > 0.5, *p* < 0.05. The threshold of co-expression network between known/novel lncRNAs and validated DEGs was with |correlation coefficient| > 0.9, *p* < 0.05.

### Statistical Analysis

Linear regression was applied to assess the correlation between validated DEGs and IVF clinical outcomes. Spearman correlation analysis was used to construct correlation heatmap between clinical characteristics and the expression of mRNAs and lncRNAs. Pearson correlation analysis was utilized to analyze co-expression relationships between mRNAs and lncRNAs. *p* < 0.05 is considered statistically significant. The data were analyzed using IBM SPSS Statistics v. 20.0 for Mac (IBM Corp., Armonk, NY, USA). Venn’s diagrams were schematized by using https://bioinfogp.cnb.csic.es/tools/venny/index.html.

## Data Availability Statement

The datasets presented in this study can be found in online repositories. The names of the repository/repositories and accession number(s) can be found below: https://bigd.big.ac.cn/gsa-human/, HRA000391.

## Ethics Statement

The studies involving human participants were reviewed and approved by the Institutional Review Board of Sir Run Run Shaw Hospital, Zhejiang University School of Medicine. The patients/participants provided their written informed consent to participate in this study.

## Author Contributions

LS made substantial contributions to conception and design and interpretation of data, and wrote and edited the manuscript. XW was involved in experimental execution and sample collection. BW and CY mainly analyzed RNA-seq data and revised the manuscript critically for important intellectual content. CL and FZ were involved in sample collection and acquisition of clinical data. YD executed qPCR to validate gene expression. JC was involved in the selection and recruitment of patients with OEM and MF. XL contributed to RNA extraction and reverse transcription. SZ devised and supervised the study and made final approval of the version to be published. All authors contributed to the article and approved the submitted version.

## Funding

This research was funded by grants from the National Natural Science Foundation of China (grant numbers 81971358, U20A20349, and 81871135), Medical Science and Technology Project Foundation of Zhejiang Province (2018KY486, 2018KY465, 2019KY411, and 2020KY606), and National Key Research and Development Program grant 2018YFC1004800.

## Conflict of Interest

The authors declare that the research was conducted in the absence of any commercial or financial relationships that could be construed as a potential conflict of interest.

## Publisher’s Note

All claims expressed in this article are solely those of the authors and do not necessarily represent those of their affiliated organizations, or those of the publisher, the editors and the reviewers. Any product that may be evaluated in this article, or claim that may be made by its manufacturer, is not guaranteed or endorsed by the publisher.

## References

[1] Bulun SE Yilmaz BD Sison C Miyazaki K Bernardi L Liu S. Endometriosis. Endocr Rev (2019) 40(4):1048–79. doi: 10.1210/er.2018-00242 PMC6693056 30994890

[2] Johnson NP Hummelshoj L Adamson GD Keckstein J Taylor HS Abrao MS. World Endometriosis Society Consensus on the Classification of Endometriosis. Hum Reprod (2017) 32(2):315–24. doi: 10.1093/humrep/dew293 27920089

[3] Younis JS Shapso N Fleming R Ben-Shlomo I Izhaki I. Impact of Unilateral Versus Bilateral Ovarian Endometriotic Cystectomy on Ovarian Reserve: A Systematic Review and Meta-Analysis. Hum Reprod Update (2019) 25(3):375–91. doi: 10.1093/humupd/dmy049 30715359

[4] Benaglia L Busnelli A Biancardi R Vegetti W Reschini M Vercellini P. Oocyte Retrieval Difficulties in Women With Ovarian Endometriomas. Reprod BioMed Online (2018) 37(1):77–84. doi: 10.1016/j.rbmo.2018.03.020 29759886

[5] Muzii L Di Tucci C Di Feliciantonio M Galati G Verrelli L Donato VD. Management of Endometriomas. Semin Reprod Med (2017) 35(1):25–30. doi: 10.1055/s-0036-1597126 27926971

[6] Shi LB Zhou F Zhu HY Huang D Jin XY Li C. Transforming Growth Factor Beta1 From Endometriomas Promotes Fibrosis in Surrounding Ovarian Tissues *via* Smad2/3 Signaling. Biol Reprod (2017) 97(6):873–82. doi: 10.1093/biolre/iox140 29136085

[7] Chapron C Marcellin L Borghese B Santulli P. Rethinking Mechanisms, Diagnosis and Management of Endometriosis. Nat Rev Endocrinol (2019) 15(11):666–82. doi: 10.1038/s41574-019-0245-z 31488888

[8] Nicolaus K Brauer D Sczesny R Jimenez-Cruz J Buhler K Hoppe I. Endometriosis Reduces Ovarian Response in Controlled Ovarian Hyperstimulation Independent of AMH, AFC, and Women's Age Measured by Follicular Output Rate (FORT) and Number of Oocytes Retrieved. Arch Gynecol Obstet (2019) 300(6):1759–65. doi: 10.1007/s00404-019-05337-z 31667607

[9] Li Y Li R Ouyang N Dai K Yuan P Zheng L. Investigating the Impact of Local Inflammation on Granulosa Cells and Follicular Development in Women With Ovarian Endometriosis. Fertil Steril (2019) 112(5):882–91.e1. doi: 10.1016/j.fertnstert.2019.07.007 31551156

[10] Gonzalez-Foruria I Soldevila PB Rodriguez I Rodriguez-Purata J Pardos C Garcia S. Do Ovarian Endometriomas Affect Ovarian Response to Ovarian Stimulation for IVF/ICSI? Reprod BioMed Online (2020) 41(1):37–43. doi: 10.1016/j.rbmo.2020.03.013 32456967

[11] Orazov MR Radzinsky VY Ivanov II Khamoshina MB Shustova VB. Oocyte Quality in Women With Infertility Associated Endometriosis. Gynecol Endocrinol (2019) 35(sup1):24–6. doi: 10.1080/09513590.2019.1632088 31532315

[12] Sanchez AM Vanni VS Bartiromo L Papaleo E Zilberberg E Candiani M. Is the Oocyte Quality Affected by Endometriosis? A Rev Lit J Ovarian Res (2017) 10(1):43. doi: 10.1186/s13048-017-0341-4 PMC5508680 28701212

[13] Choi SW Kim HW Nam JW. The Small Peptide World in Long Noncoding RNAs. Brief Bioinform (2019) 20(5):1853–64. doi: 10.1093/bib/bby055 PMC6917221 30010717

[14] Kopp F Mendell JT. Functional Classification and Experimental Dissection of Long Noncoding RNAs. Cell (2018) 172(3):393–407. doi: 10.1016/j.cell.2018.01.011 29373828 PMC5978744

[15] Cui D Ma J Liu Y Lin K Jiang X Qu Y. Analysis of Long Non-Coding RNA Expression Profiles Using RNA Sequencing in Ovarian Endometriosis. Gene (2018) 673:140–8. doi: 10.1016/j.gene.2018.06.046 29920364

[16] Ghafouri-Fard S Shoorei H Taheri M. Role of Non-Coding RNAs in the Pathogenesis of Endometriosis. Front Oncol (2020) 10:1370. doi: 10.3389/fonc.2020.01370 32850438 PMC7417625

[17] Panir K Schjenken JE Robertson SA Hull ML. Non-Coding RNAs in Endometriosis: A Narrative Review. Hum Reprod Update (2018) 24(4):497–515. doi: 10.1093/humupd/dmy014 29697794

[18] Bai J Wang B Wang T Ren W. Identification of Functional lncRNAs Associated With Ovarian Endometriosis Based on a ceRNA Network. Front Genet (2021) 12:534054. doi: 10.3389/fgene.2021.534054 33584822 PMC7873467

[19] Zhang Y Yan Z Qin Q Nisenblat V Chang HM Yu Y. Transcriptome Landscape of Human Folliculogenesis Reveals Oocyte and Granulosa Cell Interactions. Mol Cell (2018) 72(6):1021–1034 e4. doi: 10.1016/j.molcel.2018.10.029 30472193

[20] Ferrero H Corachan A Aguilar A Quinonero A Carbajo-Garcia MC Alama P. Single-Cell RNA Sequencing of Oocytes From Ovarian Endometriosis Patients Reveals a Differential Transcriptomic Profile Associated With Lower Quality. Hum Reprod (2019) 34(7):1302–12. doi: 10.1093/humrep/dez053 31211846

[21] Dumesic DA Meldrum DR Katz-Jaffe MG Krisher RL Schoolcraft WB. Oocyte Environment: Follicular Fluid and Cumulus Cells Are Critical for Oocyte Health. Fertil Steril (2015) 103(2):303–16. doi: 10.1016/j.fertnstert.2014.11.015 25497448

[22] Clarke HJ. Regulation of Germ Cell Development by Intercellular Signaling in the Mammalian Ovarian Follicle. Wiley Interdiscip Rev Dev Biol (2018) 7(1):1–22. doi: 10.1002/wdev.294 PMC5746469 28892263

[23] Li SH Lin MH Hwu YM Lu CH Yeh LY Chen YJ. Correlation of Cumulus Gene Expression of GJA1, PRSS35, PTX3, and SERPINE2 With Oocyte Maturation, Fertilization, and Embryo Development. Reprod Biol Endocrinol (2015) 13:93. doi: 10.1186/s12958-015-0091-3 26276571 PMC4537566

[24] Assidi M Montag M Sirard MA. Use of Both Cumulus Cells' Transcriptomic Markers and Zona Pellucida Birefringence to Select Developmentally Competent Oocytes in Human Assisted Reproductive Technologies. BMC Genomics (2015) 16 Suppl:1, S9. doi: 10.1186/1471-2164-16-S1-S9 25923296 PMC4315169

[25] Kahraman S Cetinkaya CP Cetinkaya M Tufekci MA Ekmekci CG Montag M. Is There a Correlation Between Follicle Size and Gene Expression in Cumulus Cells and Is Gene Expression an Indicator of Embryo Development? Reprod Biol Endocrinol (2018) 16(1):69. doi: 10.1186/s12958-018-0388-0 30031399 PMC6054838

[26] Wyse BA Fuchs Weizman N Kadish S Balakier H Sangaralingam M Librach CL. Transcriptomics of Cumulus Cells - A Window Into Oocyte Maturation in Humans. J Ovarian Res (2020) 13(1):93. doi: 10.1186/s13048-020-00696-7 32787963 PMC7425158

[27] Fragouli E Lalioti MD Wells D. The Transcriptome of Follicular Cells: Biological Insights and Clinical Implications for the Treatment of Infertility. Hum Reprod Update (2014) 20(1):1–11. doi: 10.1093/humupd/dmt044 24082041 PMC3845680

[28] Kasapoglu I Ata B Uyaniklar O Seyhan A Orhan A Yildiz Oguz S. Endometrioma-Related Reduction in Ovarian Reserve (ERROR): A Prospective Longitudinal Study. Fertil Steril (2018) 110(1):122–7. doi: 10.1016/j.fertnstert.2018.03.015 29935810

[29] Nankali A Kazeminia M Jamshidi PK Shohaimi S Salari N Mohammadi M. The Effect of Unilateral and Bilateral Laparoscopic Surgery for Endometriosis on Anti-Mullerian Hormone (AMH) Level After 3 and 6 Months: A Systematic Review and Meta-Analysis. Health Qual Life Outcomes (2020) 18(1):314. doi: 10.1186/s12955-020-01561-3 32972380 PMC7513290

[30] Ju Y Mizutani T Imamichi Y Yazawa T Matsumura T Kawabe S. Nuclear Receptor 5A (NR5A) Family Regulates 5-Aminolevulinic Acid Synthase 1 (ALAS1) Gene Expression in Steroidogenic Cells. Endocrinology (2012) 153(11):5522–34. doi: 10.1210/en.2012-1334 23024262

[31] Guo R Chen F Shi Z. Suppression of Notch Signaling Stimulates Progesterone Synthesis by Enhancing the Expression of NR5A2 and NR2F2 in Porcine Granulosa Cells. Genes (Basel) (2020) 11(2):1–12. doi: 10.3390/genes11020120 PMC7073743 31978970

[32] Bertolin K Gossen J Schoonjans K Murphy BD. The Orphan Nuclear Receptor Nr5a2 Is Essential for Luteinization in the Female Mouse Ovary. Endocrinology (2014) 155(5):1931–43. doi: 10.1210/en.2013-1765 24552399

[33] Zhang J Xu Y Liu H Pan Z. MicroRNAs in Ovarian Follicular Atresia and Granulosa Cell Apoptosis. Reprod Biol Endocrinol (2019) 17(1):9. doi: 10.1186/s12958-018-0450-y 30630485 PMC6329178

[34] Younis JS Matilsky M Radin O Ben-Ami M. Increased Progesterone/Estradiol Ratio in the Late Follicular Phase Could Be Related to Low Ovarian Reserve in *In Vitro* Fertilization-Embryo Transfer Cycles With a Long Gonadotropin-Releasing Hormone Agonist. Fertil Steril (2001) 76(2):294–9. doi: 10.1016/s0015-0282(01)01918-5 11476775

[35] Younis JS. The Role of Progesterone/Estradiol Ratio in Exploring the Mechanism of Late Follicular Progesterone Elevation in Low Ovarian Reserve Women. Med Hypotheses (2019) 125:126–8. doi: 10.1016/j.mehy.2019.02.047 30902140

[36] Griffin D Liu X Pru C Pru JK Peluso JJ. Expression of Progesterone Receptor Membrane Component-2 Within the Immature Rat Ovary and Its Role in Regulating Mitosis and Apoptosis of Spontaneously Immortalized Granulosa Cells. Biol Reprod (2014) 91(2):36. doi: 10.1095/biolreprod.114.117481 24990806 PMC4435414

[37] Peluso JJ Pru CA Liu X Kelp NC Pru JK. Progesterone Receptor Membrane Component 1 and 2 Regulate Granulosa Cell Mitosis and Survival Through a NFKappaB-Dependent Mechanismdagger. Biol Reprod (2019) 100(6):1571–80. doi: 10.1093/biolre/ioz043 PMC6561858 30877763

[38] Sueldo C Liu X Peluso JJ. Progestin and AdipoQ Receptor 7, Progesterone Membrane Receptor Component 1 (PGRMC1), and PGRMC2 and Their Role in Regulating Progesterone's Ability to Suppress Human Granulosa/Luteal Cells From Entering Into the Cell Cycle. Biol Reprod (2015) 93(3):63. doi: 10.1095/biolreprod.115.131508 26203174

[39] Skiadas CC Duan S Correll M Rubio R Karaca N Ginsburg ES. Ovarian Reserve Status in Young Women Is Associated With Altered Gene Expression in Membrana Granulosa Cells. Mol Hum Reprod (2012) 18(7):362–71. doi: 10.1093/molehr/gas008 PMC3378309 22355044

[40] Eapen MS Kota A Vindin H McAlinden KD Xenaki D Oliver BG. Apoptosis Signal-Regulating Kinase 1 Inhibition Attenuates Human Airway Smooth Muscle Growth and Migration in Chronic Obstructive Pulmonary Disease. Clin Sci (Lond) (2018) 132(14):1615–27. doi: 10.1042/CS20180398 PMC6218165 30006481

[41] Sirotkin AV Petrak J Alwasel S Harrath AH. Apoptosis Signal-Regulating Kinase (ASK1) and Transcription Factor Tumor Suppressor Protein TP53 Suppress Rabbit Ovarian Granulosa Cell Functions. Anim Reprod Sci (2019) 204:140–51. doi: 10.1016/j.anireprosci.2019.03.018 30948244

[42] Lin X Dai Y Tong X Xu W Huang Q Jin X. Excessive Oxidative Stress in Cumulus Granulosa Cells Induced Cell Senescence Contributes to Endometriosis-Associated Infertility. Redox Biol (2020) 30:101431. doi: 10.1016/j.redox.2020.101431 31972508 PMC6974790

[43] Da Broi MG de Albuquerque FO de Andrade AZ Cardoso RL Jordao Junior AA Navarro PA. Increased Concentration of 8-Hydroxy-2'-Deoxyguanosine in Follicular Fluid of Infertile Women With Endometriosis. Cell Tissue Res (2016) 366(1):231–42. doi: 10.1007/s00441-016-2428-4 27250533

[44] Sturchler E Feurstein D Chen W McDonald P Duckett D. Stress-Induced Nuclear Import of Apoptosis Signal-Regulating Kinase 1 Is Mediated by Karyopherin Alpha2/Beta1 Heterodimer. Biochim Biophys Acta (2013) 1833(3):583–92. doi: 10.1016/j.bbamcr.2012.10.023 23123190

[45] Mihalas BP Western PS Loveland KL McLaughlin EA Holt JE. Changing Expression and Subcellular Distribution of Karyopherins During Murine Oogenesis. Reproduction (2015) 150(6):485–96. doi: 10.1530/REP-14-0585 26399853

[46] Sanchez AM Vigano P Quattrone F Pagliardini L Papaleo E Candiani M. The WNT/Beta-Catenin Signaling Pathway and Expression of Survival Promoting Genes in Luteinized Granulosa Cells: Endometriosis as a Paradigm for a Dysregulated Apoptosis Pathway. Fertil Steril (2014) 101(6):1688–96. doi: 10.1016/j.fertnstert.2014.02.040 24661731

[47] Carr DW DeManno DA Atwood A Hunzicker-Dunn M Scott JD. Follicle-Stimulating Hormone Regulation of A-Kinase Anchoring Proteins in Granulosa Cells. J Biol Chem (1993) 268(28):20729–32. doi: 10.1016/S0021-9258(19)36841-3 8407895

[48] Nishimura T Sugiura K Naito K. A-Kinase Anchor Protein 1 (AKAP1) Regulates cAMP-Dependent Protein Kinase (PKA) Localization and Is Involved in Meiotic Maturation of Porcine Oocytes. Biol Reprod (2013) 88(4):85. doi: 10.1095/biolreprod.112.106351 23426434

[49] Nishimura T Fujii W Sugiura K Naito K. Cytoplasmic Anchoring of cAMP-Dependent Protein Kinase (PKA) by A-Kinase Anchor Proteins (AKAPs) Is Required for Meiotic Arrest of Porcine Full-Grown and Growing Oocytes. Biol Reprod (2014) 90(3):58. doi: 10.1095/biolreprod.113.114736 24501172

[50] Aragon L. The Smc5/6 Complex: New and Old Functions of the Enigmatic Long-Distance Relative. Annu Rev Genet (2018) 52:89–107. doi: 10.1146/annurev-genet-120417-031353 30476445

[51] Farmer S San-Segundo PA Aragon L. The Smc5-Smc6 Complex Is Required to Remove Chromosome Junctions in Meiosis. PloS One (2011) 6(6):e20948. doi: 10.1371/journal.pone.0020948 21731634 PMC3120815

[52] Hwang G Sun F O'Brien M Eppig JJ Handel MA Jordan PW. SMC5/6 Is Required for the Formation of Segregation-Competent Bivalent Chromosomes During Meiosis I in Mouse Oocytes. Development (2017) 144(9):1648–60. doi: 10.1242/dev.145607 PMC5450844 28302748

[53] Fu XH Chen CZ Li S Han DX Wang YJ Yuan B. Dual-Specificity Phosphatase 1 Regulates Cell Cycle Progression and Apoptosis in Cumulus Cells by Affecting Mitochondrial Function, Oxidative Stress, and Autophagy. Am J Physiol Cell Physiol (2019) 317(6):C1183–C93. doi: 10.1152/ajpcell.00012.2019 31532716

[54] Tu J Chen Y Li Z Yang H Chen H Yu Z. Long Non-Coding RNAs in Ovarian Granulosa Cells. J Ovarian Res (2020) 13(1):63. doi: 10.1186/s13048-020-00663-2 32503679 PMC7275442

[55] Liu H Zhang Z Xiong W Zhang L Du Y Liu Y. Long non-Coding RNA MALAT1 Mediates Hypoxia-Induced Pro-Survival Autophagy of Endometrial Stromal Cells in Endometriosis. J Cell Mol Med (2019) 23(1):439–52. doi: 10.1111/jcmm.13947 PMC6307811 30324652

[56] Feng Y Tan BZ. LncRNA MALAT1 Inhibits Apoptosis of Endometrial Stromal Cells Through miR-126-5p-CREB1 Axis by Activating PI3K-AKT Pathway. Mol Cell Biochem (2020) 475(1-2):185–94. doi: 10.1007/s11010-020-03871-y 32809092

[57] Du Y Zhang Z Xiong W Li N Liu H He H. Estradiol Promotes EMT in Endometriosis *via* MALAT1/miR200s Sponge Function. Reproduction (2019) 157(2):179–88. doi: 10.1530/REP-18-0424 PMC7305834 30500775

[58] Li Y Liu YD Chen SL Chen X Ye DS Zhou XY. Down-Regulation of Long non-Coding RNA MALAT1 Inhibits Granulosa Cell Proliferation in Endometriosis by Up-Regulating P21 *via* Activation of the ERK/MAPK Pathway. Mol Hum Reprod (2019) 25(1):17–29. doi: 10.1093/molehr/gay045 30371869

[59] Sun Z Jing C Xiao C Li T. An Autophagy-Related Long Non-Coding RNA Prognostic Signature Accurately Predicts Survival Outcomes in Bladder Urothelial Carcinoma Patients. Aging (Albany NY) (2020) 12(15):15624–37. doi: 10.18632/aging.103718 PMC7467376 32805727

[60] Xia XQ Lu WL Ye YY Chen J. LINC00662 Promotes Cell Proliferation, Migration and Invasion of Melanoma by Sponging miR-890 to Upregulate ELK3. Eur Rev Med Pharmacol Sci (2020) 24(16):8429–38. doi: 10.26355/eurrev_202008_22640 32894549

[61] Huang WJ Tian XP Bi SX Zhang SR He TS Song LY. The Beta-Catenin/TCF-4-LINC01278-miR-1258-Smad2/3 Axis Promotes Hepatocellular Carcinoma Metastasis. Oncogene (2020) 39(23):4538–50. doi: 10.1038/s41388-020-1307-3 PMC7269911 32372060

[62] Tian X Wu Y Yang Y Wang J Niu M Gao S. Long Noncoding RNA LINC00662 Promotes M2 Macrophage Polarization and Hepatocellular Carcinoma Progression *via* Activating Wnt/beta-Catenin Signaling. Mol Oncol (2020) 14(2):462–83. doi: 10.1002/1878-0261.12606 PMC6998656 31785055

[63] Tao LM Gong YF Yang HM Pei JH Zhao XJ Liu SS. LINC00662 Promotes Glycolysis and Cell Survival by Regulating miR- 375/HIF-1alpha Axis in Ovarian Cancer. J Biol Regul Homeost Agents (2020) 34(3):467–77. doi: 10.23812/19-300-A-18 32476381

[64] Joshi M Rajender S. Long non-Coding RNAs (lncRNAs) in Spermatogenesis and Male Infertility. Reprod Biol Endocrinol (2020) 18(1):103. doi: 10.1186/s12958-020-00660-6 33126901 PMC7599102

[65] Cheng B Rong A Zhou Q Li W. LncRNA LINC00662 Promotes Colon Cancer Tumor Growth and Metastasis by Competitively Binding With miR-340-5p to Regulate CLDN8/IL22 Co-Expression and Activating ERK Signaling Pathway. J Exp Clin Cancer Res (2020) 39(1):5. doi: 10.1186/s13046-019-1510-7 31900207 PMC6942292

[66] Pertea M Pertea GM Antonescu CM Chang TC Mendell JT Salzberg SL. StringTie Enables Improved Reconstruction of a Transcriptome From RNA-Seq Reads. Nat Biotechnol (2015) 33(3):290–5. doi: 10.1038/nbt.3122 PMC4643835 25690850

[67] Robinson MD McCarthy DJ Smyth GK. Edger: A Bioconductor Package for Differential Expression Analysis of Digital Gene Expression Data. Bioinformatics (2010) 26(1):139–40. doi: 10.1093/bioinformatics/btp616 PMC2796818 19910308

[68] Kong L Zhang Y Ye ZQ Liu XQ Zhao SQ Wei L. CPC: Assess the Protein-Coding Potential of Transcripts Using Sequence Features and Support Vector Machine. Nucleic Acids Res (2007) 35(Web Server issue):W345–9. doi: 10.1093/nar/gkm391 PMC1933232 17631615

[69] Sun L Luo H Bu D Zhao G Yu K Zhang C. Utilizing Sequence Intrinsic Composition to Classify Protein-Coding and Long Non-Coding Transcripts. Nucleic Acids Res (2013) 41(17):e166. doi: 10.1093/nar/gkt646 23892401 PMC3783192

[70] Walter W Sanchez-Cabo F Ricote M. GOplot: An R Package for Visually Combining Expression Data With Functional Analysis. Bioinformatics (2015) 31(17):2912–4. doi: 10.1093/bioinformatics/btv300 25964631

[71] Ye Z Zeng Z Wang D Lei S Shen Y Chen Z. Identification of Key Genes Associated With the Progression of Intrahepatic Cholangiocarcinoma Using Weighted Gene Co-Expression Network Analysis. Oncol Lett (2020) 20(1):483–94. doi: 10.3892/ol.2020.11600 PMC7286119 32565973

