# Supplementary material for: Molecular Signatures Correlated With Poor IVF Outcomes: Insights From the mRNA and lncRNA Expression of Endometriotic Granulosa Cells
Source: Front Endocrinol (Lausanne). 2022 Feb 28;13:825934. doi: 10.3389/fendo.2022.825934 (PMC8919698; doi:10.3389/fendo.2022.825934)
Supplement: Supplementary file 1 [file DataSheet_1.zip › Supplemental Figures S1-S4.DOCX]

Supplementary Material


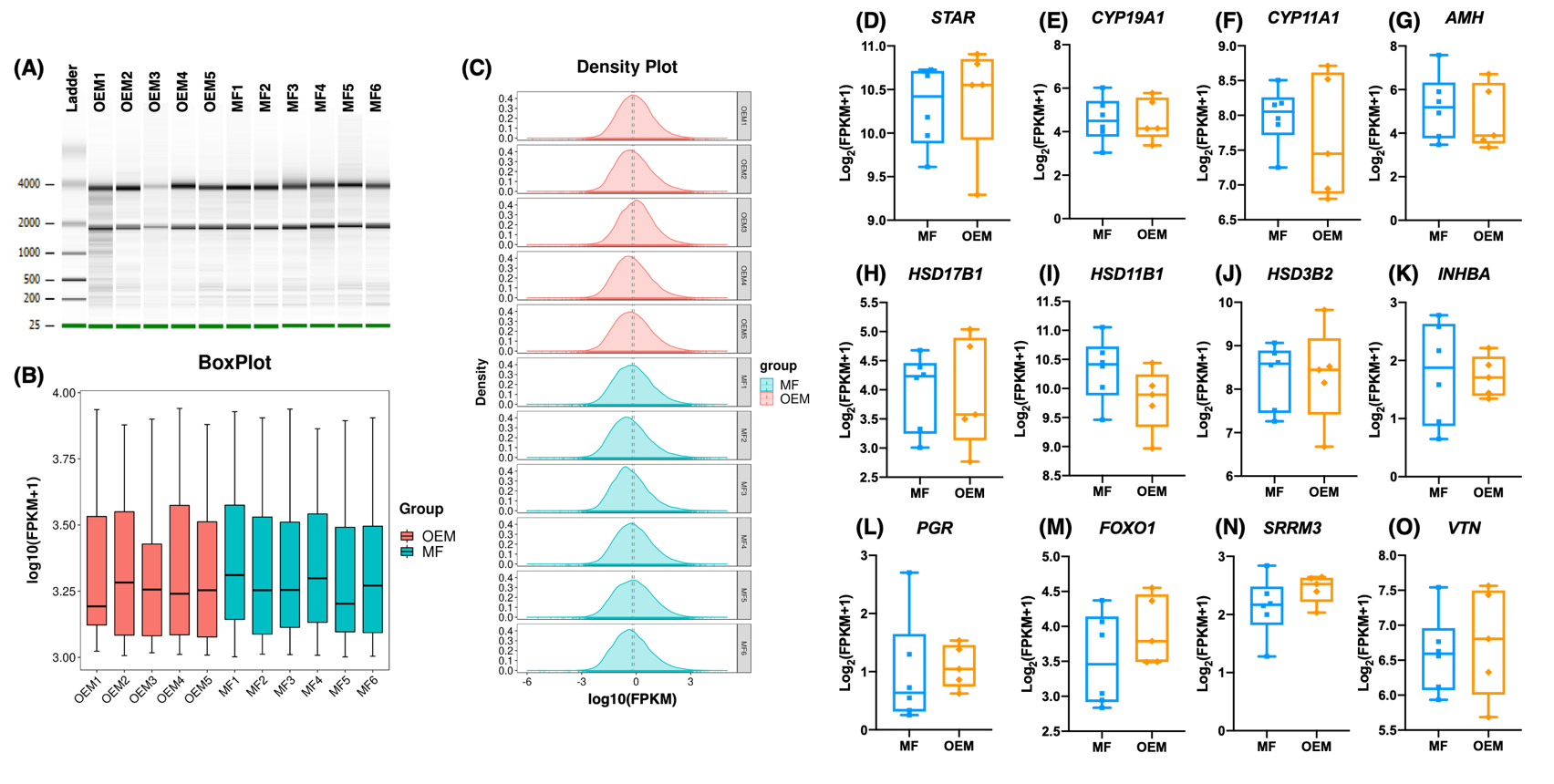
**Supplemental Figure S1**

**Figure S1.** RNA-seq sample quality inspection and overall gene expression level analysis of RNA-seq data. (A) Simulated RNA electrophoretic map according to the peak diagram of 2100 Bioanalyzer quality inspections shows that all the RNA bands were complete and clear without diffusion. (B) This statistical chart of the gene expression value distribution of each sample demonstrates the gene expression level compared with the overall level. The abscissa is the sample name, and the ordinate is log_10_(FPKM+1). (C) The gene expression density maps for each sample conform to the normal distribution, and the expression trend is consistent. The abscissa is log_10_(FPKM), and the ordinate is the gene expression density. (D-O) The fragments per kilobase of exons per million mapped reads (FPKM) values of cell-type-specific markers of GCs between the OEM and MF groups.


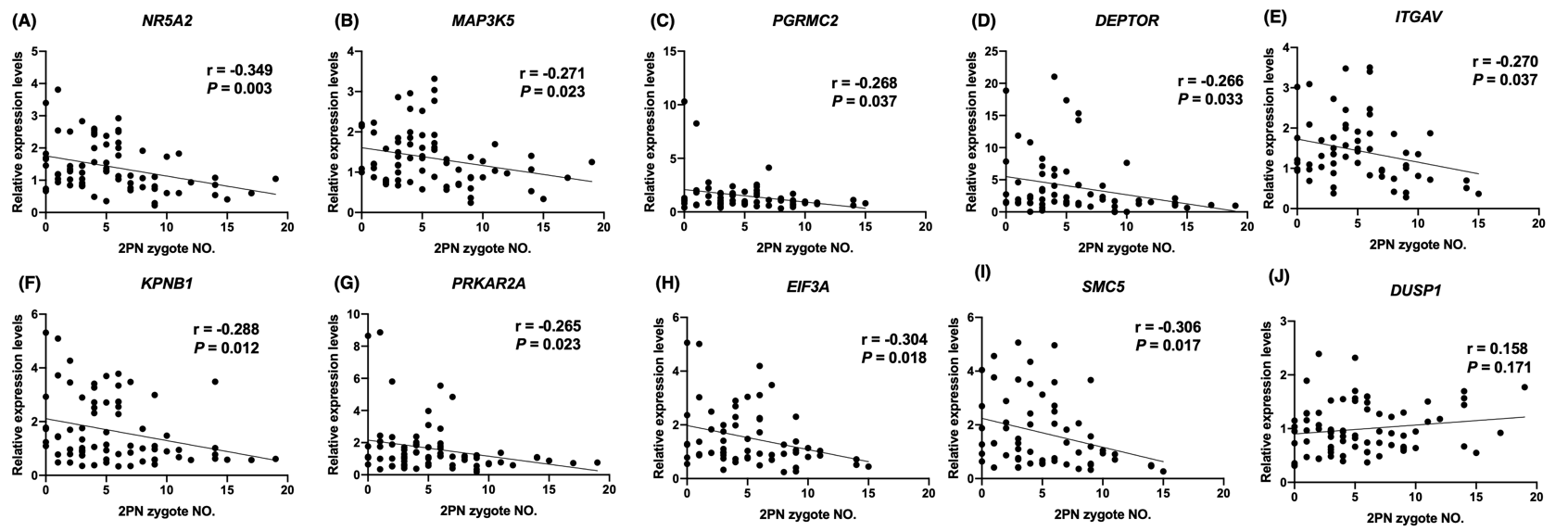
**Supplemental Figure S2**

**Figure S2.** Correlation analysis between relative expression levels of the validated DEGs and the numbers of 2PN zygotes (A–J). (*r*, correlation coefficient)

**Supplemental Figure S3**


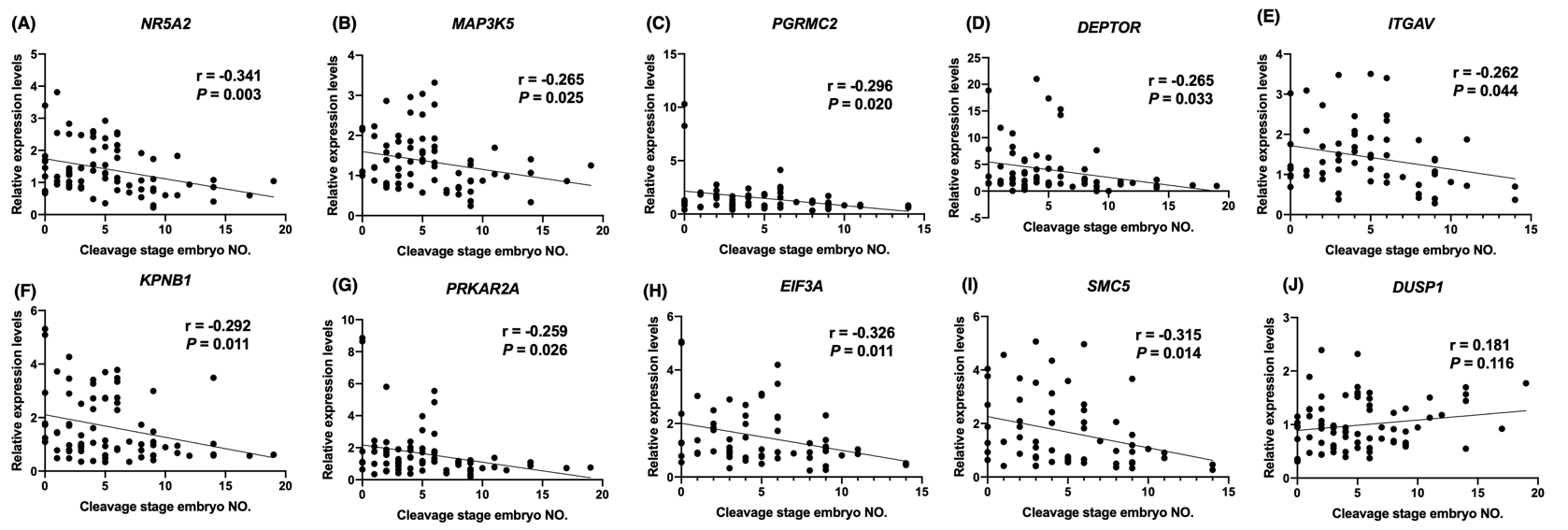


**Figure S3.** Correlation analysis between relative expression levels of the validated DEGs and the numbers of cleavage stage embryos (A–J). (*r*, correlation coefficient)


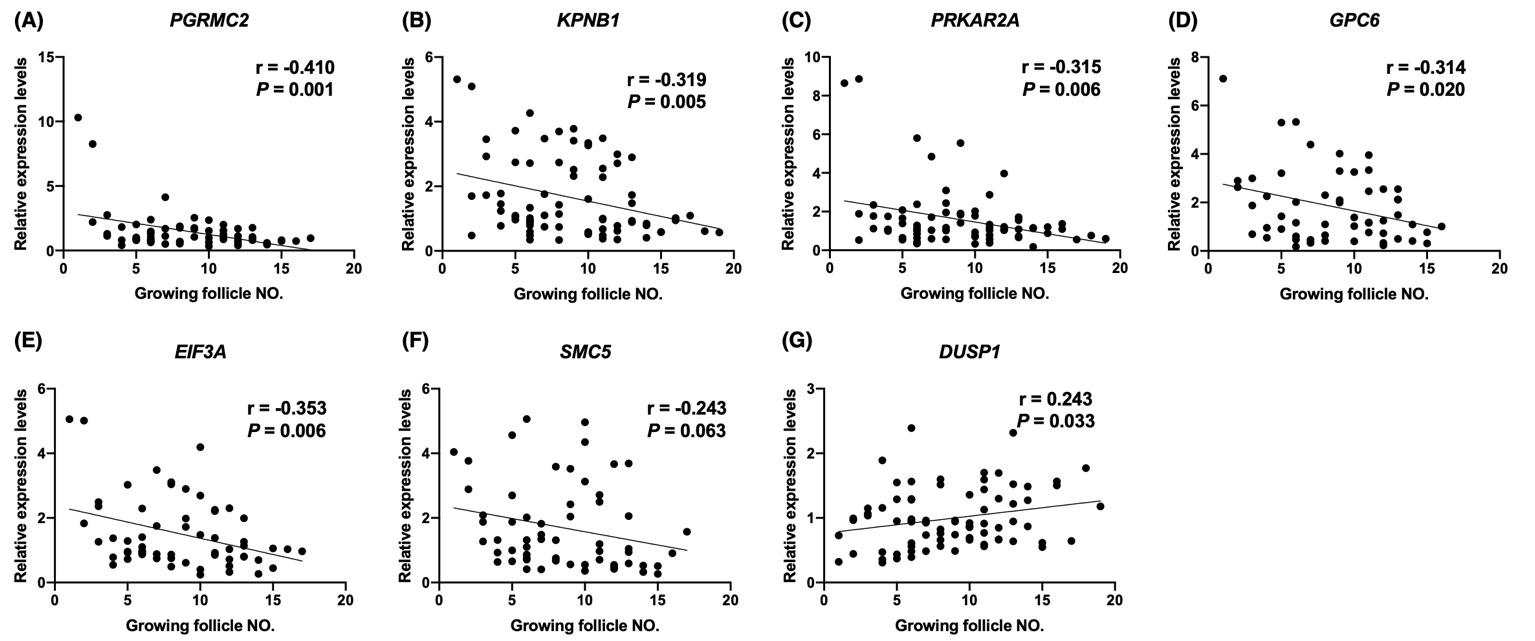
**Supplemental Figure S4**

**Figure S4.** Correlation analysis between relative expression levels of the validated DEGs and the growing follicle numbers (A–G). (*r*, correlation coefficient)
