# Supplementary material for: Molecular Signatures Correlated With Poor IVF Outcomes: Insights From the mRNA and lncRNA Expression of Endometriotic Granulosa Cells
Source: Front Endocrinol (Lausanne). 2022 Feb 28;13:825934. doi: 10.3389/fendo.2022.825934 (PMC8919698; doi:10.3389/fendo.2022.825934)
Supplement: Supplementary file 1 [file DataSheet_1.zip › Supplemental Tables S1, S2, S6, S7.DOCX]

Supplementary Material

**Supplemental Table S1.** Quality control statistics for the RNA-sequencing.

| Sample | Raw Data | | | Valid Data | | | Valid Ratio%(reads) | Q20% | Q30% | GC content% |
| --- | --- | --- | --- | --- | --- | --- | --- | --- | --- | --- |
|  | Read | Base | | Read | Base | |  |  |  |  |
| OEM1 | 77744666 | | 11.66G | 36077926 | | 5.41G | 46.41 | 99.96 | 97.13 | 49 |
| OEM2 | 52741588 | | 7.91G | 31198420 | | 4.68G | 59.15 | 99.97 | 97.16 | 51 |
| OEM3 | 51897816 | | 7.78G | 31497220 | | 4.72G | 60.69 | 99.96 | 96.37 | 47 |
| OEM4 | 55631934 | | 8.34G | 36368988 | | 5.46G | 65.37 | 99.93 | 95.42 | 49 |
| OEM5 | 76279306 | | 11.44G | 40582732 | | 6.09G | 53.20 | 99.97 | 97.13 | 50 |
| Ctrl1 | 74698708 | | 11.20G | 43692548 | | 6.55G | 58.49 | 99.97 | 97.21 | 50 |
| Ctrl2 | 89652840 | | 13.45G | 46615244 | | 6.99G | 52.00 | 99.96 | 96.60 | 51 |
| Ctrl3 | 64267452 | | 9.64G | 37604448 | | 5.64G | 58.51 | 99.95 | 96.24 | 50.50 |
| Ctrl4 | 96403274 | | 14.46G | 37274996 | | 5.59G | 38.67 | 99.96 | 97.55 | 51 |
| Ctrl5 | 93390658 | | 14.01G | 47382066 | | 7.11G | 50.74 | 99.97 | 97.51 | 50.50 |
| Ctrl6 | 72719182 | | 10.91G | 37670120 | | 5.65G | 51.80 | 99.97 | 97.21 | 50 |

Table parameter description:

Raw Data Reads: the number of reads of the original offline data

Valid Data Reads: number of valid data reads

Valid Ratio%: Proportion of effective reads

Base: Data size

Q20%: proportion of bases with Q20% quality value ≥ 20 (sequencing error rate less than 0.01)

Q30%: proportion of bases with Q30% quality value ≥ 30 (sequencing error rate less than 0.001)

GC content%: the proportion of GC content

**Supplemental Table S2.** RNA quality detection for RNA-seq samples.

| Sample  ID | Isolation  Method | O.D.  260/280 | O.D.  260/230 | Conc.  (ng/µL) | Amount  (ng) | QC  Evaluation |
| --- | --- | --- | --- | --- | --- | --- |
| OEM1 | Trizol+DP431 | 2.20 | 0.38 | 5.82 | 128.04 | Qualified |
| OEM2 | Trizol+DP431 | 2.07 | 0.43 | 18.09 | 397.98 | Qualified |
| OEM3 | Trizol+DP431 | 1.71 | 0.31 | 0.92 | 20.24 | Qualified |
| OEM4 | Trizol+DP431 | 1.87 | 0.59 | 11.20 | 246.40 | Qualified |
| OEM5 | Trizol+DP431 | 1.90 | 0.45 | 5.13 | 112.86 | Qualified |
| Ctrl1 | Trizol+DP431 | 1.78 | 0.30 | 3.63 | 79.86 | Qualified |
| Ctrl2 | Trizol+DP431 | 2.05 | 1.31 | 39.80 | 875.60 | Qualified |
| Ctrl3 | Trizol+DP431 | 2.07 | 1.00 | 90.20 | 1,984.40 | Qualified |
| Ctrl4 | Trizol+DP431 | 1.82 | 0.16 | 9.33 | 205.26 | Qualified |
| Ctrl5 | Trizol+DP431 | 1.74 | 0.09 | 1.83 | 40.26 | Qualified |
| Ctrl6 | Trizol+DP431 | 2.17 | 1.47 | 22.28 | 490.16 | Qualified |

**Supplemental Table S6.** Endometriotic information of ovarian endometriomas (OEM) patients

| Case NO. | Age (year) | Endometriotic info about surgery | | | |  | | Endometriotic info during IVF treatment | | | | |
| --- | --- | --- | --- | --- | --- | --- | --- | --- | --- | --- | --- | --- |
|  |  | Distribution | Location | Surgery date | Stage |  | Distribution | | Location | Size (cm) | GCs obtain date |  |
| RNA-seq-1# | 27 | Unilateral | Right ovary | May 2017 | null |  | Unilateral | | Left ovary | 1.7 | Jan. 2019 |  |
| RNA-seq-2# | 30 | Bilateral | / | Sep. 2015 | IV |  | / | | / | / | Mar. 2019 |  |
| RNA-seq-3# | 30 | Bilateral | / | Oct. 2017 | IV |  | / | | / | / | May 2018 |  |
| RNA-seq-4# | 32 | Bilateral | / | Jan. 2019 | IV |  | / | | / | / | Apr. 2019 |  |
| RNA-seq-5# | 26 | Bilateral | / | Oct. 2017 | IV |  | Unilateral | | Right ovary | 1.74*1.23 | Feb. 2019 |  |
| Validation-1# | 30 | Bilateral | / | Jun. 2017 | II |  | / | | / | / | May 2018 |  |
| Validation-2# | 30 | Bilateral | / | Feb. 2018 | IV |  | / | | / | / | Jun. 2018 |  |
| Validation-3# | 26 | Bilateral | / | Oct. 2017 | IV |  | / | | / | / | Dec. 2018 |  |
| Validation-4# | 31 | Bilateral | / | Oct. 2018 | IV |  | / | | / | / | Dec. 2018 |  |
| Validation-5# | 30 | Bilateral | / | Sep. 2018 | IV |  | / | | / | / | Jan. 2019 |  |
| Validation-6# | 35 | Bilateral | / | Oct. 2017 | IV |  | / | | / | / | Jan. 2019 |  |
| Validation-7# | 28 | Bilateral | / | Feb. 2017 – right ovary;  Nov. 2018 - left ovary | III |  | Unilateral | | Left ovary | 2.87*4.7 | Feb. 2019 |  |
| Validation-8# | 29 | Unilateral | Right ovary | Jun. 2018 | III |  | Unilateral | | Right ovary | 2 | Mar. 2019 |  |
| Validation-9# | 29 | Bilateral | / | Jun. 2017 | null |  | / | | / | / | Mar. 2019 |  |
| Validation-10# | 31 | Bilateral | / | Nov. 2018 | IV |  | / | | / | / | Mar. 2019 |  |
| Validation-11# | 28 | Bilateral | / | Aug. 2018 | IV |  | / | | / | / | Mar. 2019 |  |
| Validation-12# | 37 | Unilateral | Right ovary | Dec. 2018 | null |  | / | | / | / | Mar. 2019 |  |
| Validation-13# | 33 | Bilateral | / | May 2016 | null |  | / | | / | / | Mar. 2019 |  |
| Validation-14# | 29 | Unilateral | Left ovary | Dec. 2018 | null |  | / | | / | / | Mar. 2019 |  |
| Validation-15# | 32 | Bilateral | / | Dec. 2018 | IV |  | / | | / | / | Mar. 2019 |  |
| Validation-16# | 36 | Unilateral | Left ovary | 2008 | III |  | / | | / | / | Mar. 2019 |  |
| Validation-17# | 32 | Bilateral | / | Jan. 2019 | III |  | / | | / | / | Mar. 2019 |  |
| Validation-18# | 37 | Bilateral | / | 2013 | null |  | Unilateral | | Right ovary | 1.5; 0.9 | Mar. 2019 |  |
| Validation-19# | 28 | Bilateral | / | Oct. 2014 | null |  | Unilateral | | Right ovary | 3.9*4.7; 1.7*2.0 | Mar. 2019 |  |
| Validation-20# | 36 | Unilateral | Left ovary | Apr. 2018 | III |  | / | | / | / | Apr. 2019 |  |
| Validation-21# | 40 | Bilateral | / | 2012 | null |  | / | | / | / | Apr. 2019 |  |
| Validation-22# | 33 | Bilateral | / | Dec. 2017 | null |  | / | | / | / | Apr. 2019 |  |
| Validation-23# | 41 | Bilateral | / | 2014 | null |  | / | | / | / | Apr. 2019 |  |
| Validation-24# | 35 | Unilateral | Left ovary | Jun. 2017 | II |  | / | | / | / | Apr. 2019 |  |
| Validation-25# | 29 | Unilateral | Left ovary | Dec. 2017 | III |  | / | | / | / | Apr. 2019 |  |
| Validation-26# | 27 | Unilateral | Right ovary | Aug. 2016 | null |  | / | | / | / | Apr. 2019 |  |
| Validation-27# | 40 | Unilateral | Right ovary | 2013 | null |  | / | | / | / | Apr. 2019 |  |
| Validation-28# | 34 | Unilateral | Left ovary | Mar. 2018 | III |  | / | | / | / | Apr. 2019 |  |
| Validation-29# | 41 | Unilateral | Left ovary | 2001 | null |  | / | | / | / | Apr. 2019 |  |
| Validation-30# | 35 | Bilateral | / | Apr. 2015 | III |  | Unilateral | | Left ovary | 2.69*2.06 | Jun. 2016 |  |
| Validation-31# | 37 | Unilateral | Left ovary | Apr. 2015 | IV |  | / | | / | / | Jun. 2016 |  |
| Validation-32# | 31 | Bilateral | / | Sep. 2016 | null |  | / | | / | / | May 2018 |  |
| Validation-33# | 42 | Unilateral | Left ovary | Mar. 2018 | IV |  | / | | / | / | May 2018 |  |
| Validation-34# | 30 | Unilateral | Right ovary | Jan. 2017 | III |  | / | | / | / | May 2018 |  |
| Validation-35# | 29 | Unilateral | Left ovary | 2012 | null |  | / | | / | / | May 2018 |  |
| Validation-36# | 28 | Unilateral | Left ovary | Apr. 2018 | IV |  | / | | / | / | Jun. 2018 |  |
| Validation-37# | 33 | Unilateral | Left ovary | Jan. 2018 | null |  | / | | / | / | Jun. 2018 |  |
| Validation-38# | 26 | Unilateral | Right ovary | 2012 | null |  | / | | / | / | Nov. 2018 |  |
| Validation-39# | 35 | Bilateral | / | 2019 | IV |  | / | | / | / | Apr. 2020 |  |
| Validation-40# | 36 | Unilateral | Left ovary | 2017 | III |  | / | | / | / | Jun. 2020 |  |
| Validation-41# | 31 | Bilateral | / | Aug. 2019 | null |  | Unilateral | | Left ovary | 3.74*2.69; 2.74*2.58 | Jun. 2020 |  |
| Validation-42# | 31 | Unilateral | Right ovary | May 2019 | III |  | / | | / | / | Jun. 2020 |  |

Note:

Null represents missing data due to the loss of other hospitals’ surgery records.

**Supplemental Table S7.** Primer sequences designed for differentially expressed genes validation through qPCR.

| **Primers** | **DNA sequence** | | |  |
| --- | --- | --- | --- | --- |
| *GAPDH* | Forward sequence | 5’-CATGAGAAGTATGACAACAGCCT-3’ | | |
|  | Reverse sequence | | 5’-AGTCCTTCCACGATACCAAAGT-3’ |  |
| *NR5A2* | Forward sequence | | 5’-AGCCACCCTCAACAACCTCA-3’ |  |
|  | Reverse sequence | | 5’-GCACCAAGAATTTCAGACATACGA-3’ |  |
| *MAP3K5* | Forward sequence | | 5’-GAGAGCCTGTGCTAACGACT-3’ |  |
|  | Reverse sequence | | 5’-TGATCCAGCTGAAAGAGCTGAAA-3’ |  |
| *PGRMC2* | Forward sequence | | 5’-ATGGAGAGTGTTCGAGAATGGG-3’ |  |
|  | Reverse sequence | | 5’-TCTGAAGGCCCCTGACTTTG-3’ |  |
| *DEPTOR* | Forward sequence | | 5’-GTCTGTGAGGGCAGACTGAT-3’ |  |
|  | Reverse sequence | | 5’-AACCTTTTCTTCGTGCAGCCT-3’ |  |
| *ITGAV* | Forward sequence | | 5’-TCGGATTTTCTGTAGCTGCC-3’ |  |
|  | Reverse sequence | | 5’-TCTGTAGAGACACTGAGACCTG-3’ |  |
| *KPNB1* | Forward sequence | | 5’-GAGAAGACCGTGTCTCCCGAT-3’ |  |
|  | Reverse sequence | | 5’-GATTTGCCAGCACTCTGGAC-3’ |  |
| *PRKAR2A* | Forward sequence | | 5’-CGGGCAGTAGCATGGGGAAT-3’ |  |
|  | Reverse sequence | | 5’-CAACACATGCTCCTCTCCATGA-3’ |  |
| *GPC6* | Forward sequence | | 5’-ATCGGGGCTGTGATTCTTCC-3’ |  |
|  | Reverse sequence | | 5’-CATTTCTGTGGTGCAGCATGT-3’ |  |
| *EIF3A* | Forward sequence | | 5’-ACAGGCAGTGTTTGGACCTT-3’ |  |
|  | Reverse sequence | | 5’-CTTTGGTTATGGTGGCGCTG-3’ |  |
| *SMC5* | Forward sequence | | 5’-AAGCAAAAAGGCCATGGGTG-3’ |  |
|  | Reverse sequence | | 5’-TGCATTTTTGAGATGCCTCCTT-3’ |  |
| *DUSP1* | Forward sequence | | 5’- TCGAGAGGGCTGGTCCTTAT-3’ |  |
|  | Reverse sequence | | 5’- TTGGTCCCGAATGTGCTGAG-3’ |  |

Abbreviations: DEGs, differentially expressed genes; qPCR, real time quantitative polymerase chain reaction.
